# 

**Published:** 1877-05

**Authors:** 


We call particular attention to the following notices:

"A meeting of the Provisional Association of American Medical Colleges will be held at the Palmer House, Chicago, on Saturday, June 2d, 1877, at 10 o'clock, A. M. All colleges represented at the meeting of the Association, held June, 1876, are invited to send delegates to the ensuing meeting, and all chartered medical colleges in the United States recognized as "regular" by the colleges already represented in this Association, are also invited to send delegates from their faculties to the said meeting. J. B. BIDDLE, M.D., President."

"The Annual Meeting of The Association of American Medical Editors will be held at the Palmer House, Chicago, on Monday evening, June 4th, 1877, at 7.30 o'clock.

"All medical editors are eligible for membership, and are cordially requested to be present and participate in the meeting. F. H. DAVIS, Secretary."

---

DR. SUSSDORF, formerly of Macon, Ga., has removed to New York, where he proposes to practice in future. While we are satisfied that we have lost one very eminent physician, we congratulate our friends of the metropolis on the acquisition of one more star to their already resplendent galaxy of medical talent.

The following have been received, but, for want of space, are reserved for future notice:

Contributions to Reparative Surgery. Showing its application to the treatment of deformities, produced by destructive disease or injury; congenital defects, from arrest, or excess of development; and cicatricial contractions, from burns. By Gurdon Buck, M. D.. Illustrated by numerous engravings. D. Appleton & Co., publishers. New York, 1876.

Transactions of the Pathological Society of Philadelphia. Vol. V. Containing the report of the proceedings for the year 1874, and from January, 1875, to July, 1875, edited by James Tyson, M. D., Hospital Professor of Pathological Anatomy and Histology, in the University of Pennsylvania, etc., Recorder of the Society. Printed for the Society, by J. B. Lippincott & Co., Philadelphia, 1876.

Transactions of the American Gynæcological Society, for the year 1876. Vol. I. H. O. Houghton & Co., publishers. Boston, 1877.

Solution and Absorption of Medicines; or, the best means of securing the good effects of medicines in the cure of disease. A paper read before the Tri-States Medical Society, at Vincennes, Ind., November 22, 1876. By J. W. Compton, M. D., Professor of Materia Medica and Therapeutics, in the Medical College of Evansville, Indiana.

